# Integrative Multiomics Nominate GGCT as a Crucial Regulator of Immunosuppression in Colorectal Cancer

**DOI:** 10.1155/ijog/7013449

**Published:** 2026-01-21

**Authors:** Qichao Niu, Yang Liu, Kejin Huang, Lisheng Nie, Shiming Zhao, Shifeng Yang, Changlei Su

**Affiliations:** ^1^ Department of General Surgery, Second Affiliated Hospital of Harbin Medical University, Harbin, China, hrbmush.edu.cn; ^2^ Department of Gynecology, Tumor Hospital of Harbin Medical University, Harbin, China

## Abstract

Colorectal cancer (CRC) remains a leading cause of cancer‐related mortality worldwide, with tumor microenvironment (TME) heterogeneity playing a critical role in disease progression and therapeutic response. Immune escape (IE) mechanisms facilitate tumor evasion from host immune surveillance, yet their characterization at the single‐cell level in CRC is incomplete. This study integrated single‐cell RNA sequencing (scRNA‐seq) and bulk transcriptomic data from multiple public cohorts to systematically explore IE‐related signatures in CRC. We identified major and minor cell populations within the TME and performed differential gene expression analysis. Using high‐dimensional weighted gene coexpression network analysis (hdWGCNA), we identified gene modules correlated with IE activity. Subsequent survival analysis across six independent cohorts revealed Gamma‐glutamylcyclotransferase (GGCT) as a novel prognostic biomarker associated with poor survival. Functional enrichment analysis indicated GGCT′s involvement in critical oncogenic pathways. Furthermore, GGCT expression correlated with altered immune infiltration profiles and stromal components, suggesting its role in modulating the immunosuppressive TME. Additionally, GGCT demonstrated potential predictive value for response to immunotherapy across multiple datasets. Our findings highlight GGCT as a key player in CRC immune evasion and a promising therapeutic target.

## 1. Introduction

Colorectal cancer (CRC) is one of the most prevalent malignancies globally, accounting for significant morbidity and mortality [1, 2]. Despite advances in early detection and treatment, metastatic CRC continues to pose therapeutic challenges, with resistance to conventional therapies and immunotherapy being common [3–5]. A deeper understanding of the molecular mechanisms that drive CRC progression is essential for developing novel therapeutic strategies [6–8]. The tumor microenvironment (TME) is a complex ecosystem comprising tumor cells, immune cells, stromal cells, and extracellular matrix components, all of which interact to influence tumor behavior and response to treatment. In particular, the immune contexture of the TME is a critical determinant of patient prognosis and therapeutic efficacy.

Immune escape (IE) is a hallmark of cancer, enabling tumor cells to evade detection and destruction by the immune system [9]. Mechanisms of IE include downregulation of antigen presentation, recruitment of immunosuppressive cells, and expression of immune checkpoint molecules [10]. Although these mechanisms have been studied in various cancers, the specific gene expression programs underlying IE in CRC at the single‐cell resolution remain poorly characterized. A deeper understanding of these programs could identify novel biomarkers and therapeutic targets [11, 12]. Recent advances in single‐cell RNA sequencing (scRNA‐seq) have allowed for unprecedented resolution in dissecting the cellular heterogeneity of the TME [13]. Integrating scRNA‐seq data with bulk transcriptomic data from multiple cohorts can enhance the robustness of identifying consistently prognostic genes.

In this study, we leveraged scRNA‐seq data from public repositories to first identify and characterize the cellular composition of the CRC TME. We then focused on a set of 182 previously identified IE‐related genes to quantify IE activity at the single‐cell level. Using hdWGCNA, we constructed coexpression networks to identify gene modules most associated with this IE signature. Through rigorous survival analysis across six independent CRC cohorts, we pinpointed GGCT as a top prognostic gene. We further explored its biological functions, its correlation with immune infiltration, and its predictive value for immunotherapy response. Our comprehensive analysis is aimed at providing new insights into the mechanisms of immune evasion in CRC and to propose GGCT as a potential target for future therapeutic strategies.

## 2. Materials and Methods

### 2.1. Data Collection and Processing

We obtained single‐cell transcriptomic data from GSE132465 and GSE144735 [14]. The R package Seurat was applied to identify major cell types [15]. We obtained the transcriptome data and corresponding clinical information of CRC patients from six publicly available cohorts, including COADREAD_TCGA [16], GSE106584 [17], GSE29621 [18], GSE103479 [19], GSE17538 [20], and GSE72969 [21]. The R package sva was used to remove the batch effect.

### 2.2. Bioinformatics Analysis

The 182 IE‐related genes were obtained from the previous finding [22]. The IE signature was calculated in each cell using the R package AUCell. The R package hdWGCNA was used to identify the module genes related to the IE signature [23]. The Kaplan–Meier survival curves for overall survival (OS) were plotted and compared using the R package survminer. Drug sensitivity to GGCT was predicted using oncoPredict [24]. The immune infiltration characteristics related to GGCT were predicted using the tumor immune estimation resource (TIMER) [25]. The connection between GGCT and stromal score, immune score, ESTIMATE score, and tumor purity was analyzed using the ESTIMATE algorithm [26]. The Metascape platform was employed for pathway annotation of GGCT [27].

## 3. Results

### 3.1. Cellular Heterogeneity of the CRC TME Revealed by scRNA‐seq

We began by analyzing scRNA‐seq data from GEO datasets (GSE132465 and GSE144735) using the Seurat pipeline. This analysis identified major cell types within the CRC TME, including epithelial cells, T cells, B cells, myeloid cells, fibroblasts, and endothelial cells (Figure 1a). Further subclustering revealed minor cell populations, such as distinct CD4+ T cell subsets, plasma cells, and macrophage subtypes (Figure 1b). We also successfully identified malignant epithelial cells based on copy number variation inference and marker gene expression (Figure 1c). This detailed atlas provided the foundation for subsequent cell type–specific analyses.

Figure 1Identification of microenvironment cells at the scRNA‐seq level of CRC. (a) Major cell types within the microenvironment of CRC. (b) Minor cell types within the microenvironment of CRC. (c) Tumor cells within the microenvironment of CRC.

**Figure:**
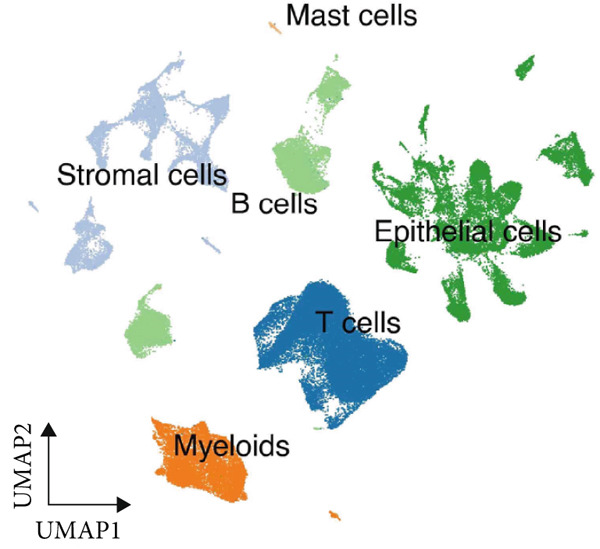
(a)

**Figure:**
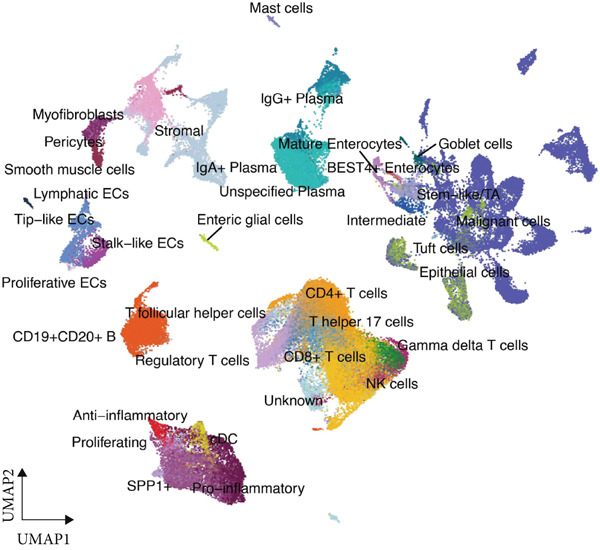
(b)

**Figure:**
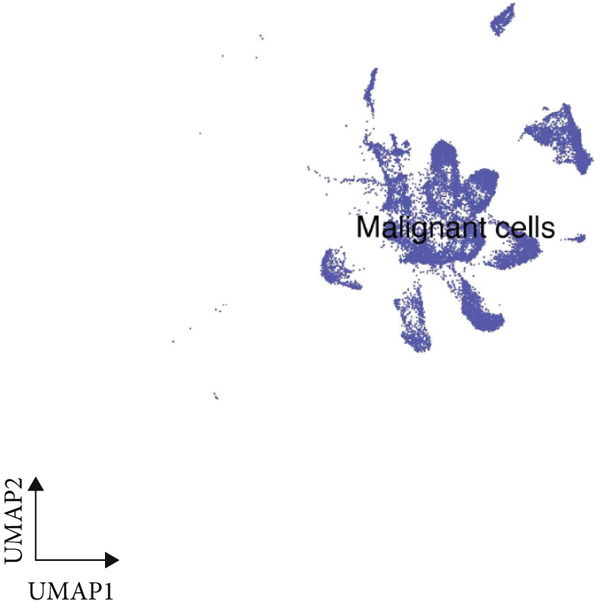
(c)

### 3.2. Differential Gene Expression Across TME Cell Types

**Figure 2:**
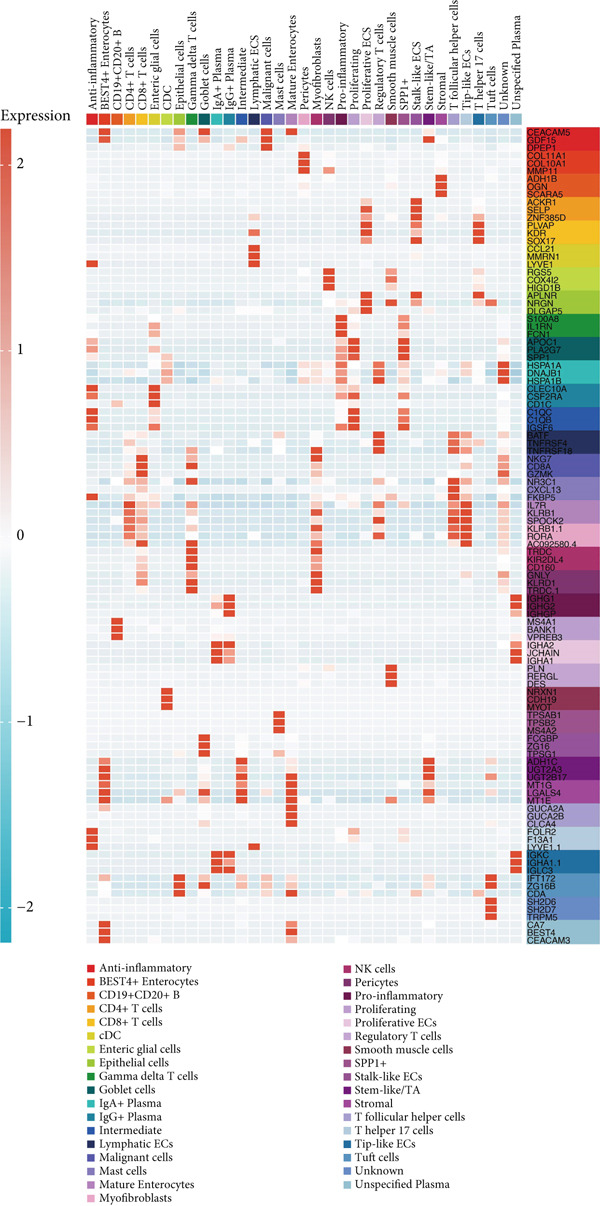
Identification of DEGs among microenvironment cells at the scRNA‐seq level of CRC.

Differential expression analysis between the major cell types identified numerous significantly dysregulated genes. Figure 2 highlights the top differentially expressed genes (DEGs) distinguishing immune cells from stromal and tumor cells. Notably, genes involved in antigen presentation (e.g., HLA genes) were highly expressed in myeloid cells, whereas genes related to matrix remodeling (e.g., COL1A1) were upregulated in fibroblasts.

### 3.3. Identification of IE‐Related Gene Coexpression Modules via hdWGCNA

To investigate the coordination of IE‐related genes, we performed hdWGCNA on the scRNA‐seq data. We first determined an appropriate soft power threshold based on scale‐free topology fit and connectivity metrics (Figure 3a). The analysis identified several coexpressed gene modules, with the dendrogram illustrating the grouping of genes into distinct modules (Figure 3b). Correlation analysis between module eigengenes and the IE signature score (calculated by AUCell) revealed that the yellow module was most positively correlated with IE activity (Figure 3c), suggesting its strong involvement in immune evasion processes.

Figure 3hdWGCNA on IE‐related gene module. (a) The association between soft power threshold and scale‐free topology model fit and mean/median/max connectivity. (b) hdWGCNA dendrogram showing the distribution of gene modules. (c) Heatmap showing the correlation between IE signature and gene modules.

**Figure:**
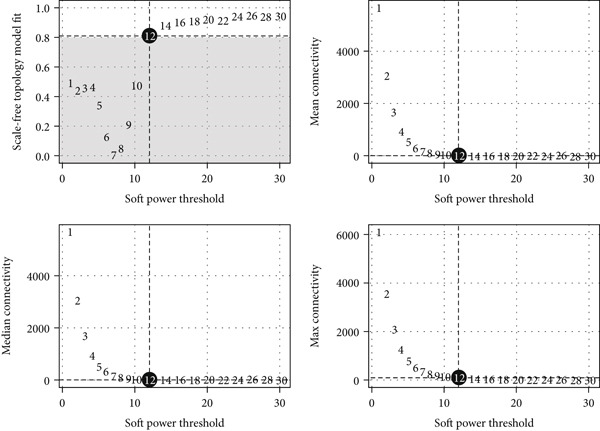
(a)

**Figure:**
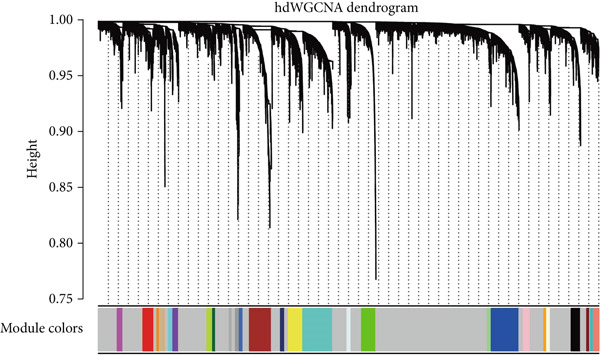
(b)

**Figure:**
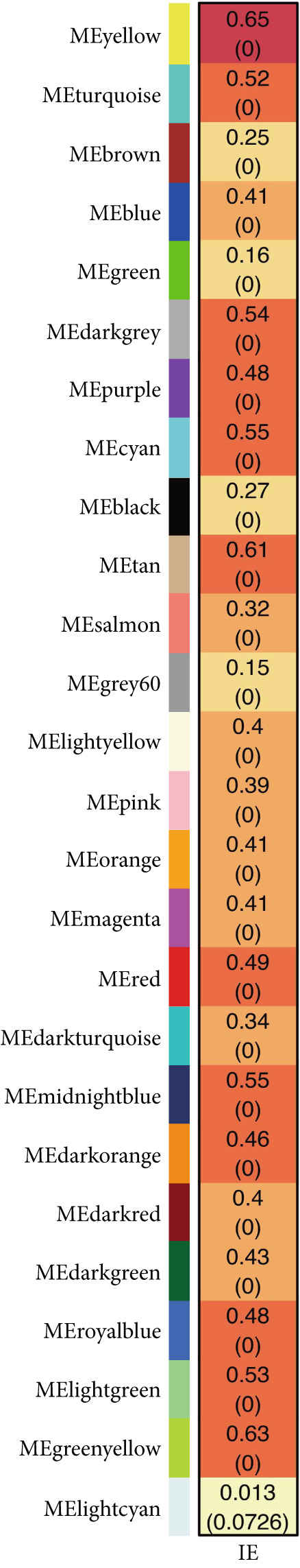
(c)

### 3.4. GGCT Is a Prognostic Biomarker in Multiple CRC Cohorts

The yellow module from hdWGCNA contained numerous genes. Univariate Cox regression analysis performed on these genes across the TCGA COADREAD cohort identified several significant prognostic genes, with GGCT being among the top hazards (hazard ratio > 1, *p* < 0.001) (Figure 4a). Examination of GGCT expression at the single‐cell level showed its predominant expression in malignant epithelial cells and a subset of myeloid cells (Figure 4b). We then stratified patients in each of the six bulk transcriptomic cohorts (TCGA, GSE106584, GSE29621, GSE103479, GSE17538, and GSE72969) into high and low GGCT expression groups. Kaplan–Meier survival analysis consistently demonstrated that high GGCT expression was significantly associated with worse OS across all cohorts (Figures 4c, 4d, 4e, 4f, 4g, and 4h, all log–rank *p* < 0.05), solidifying its role as a robust adverse prognostic marker.

Figure 4Identification of GGCT as a hazardous marker in CRC. (a) Univariate Cox regression analysis on module genes from the yellow module. (b) Vlnplot showing the GGCT expression in microenvironment cells. (c) Survival curves of GGCT‐based groups in the TCGA cohort. (d) Survival curves of GGCT‐based groups in the GSE103479 cohort. (e) Survival curves of GGCT‐based groups in the GSE106584 cohort. (f) Survival curves of GGCT‐based groups in the GSE17538 cohort. (g) Survival curves of GGCT‐based groups in the GSE29621 cohort. (h) Survival curves of GGCT‐based groups in the GSE72969 cohort.

**Figure:**
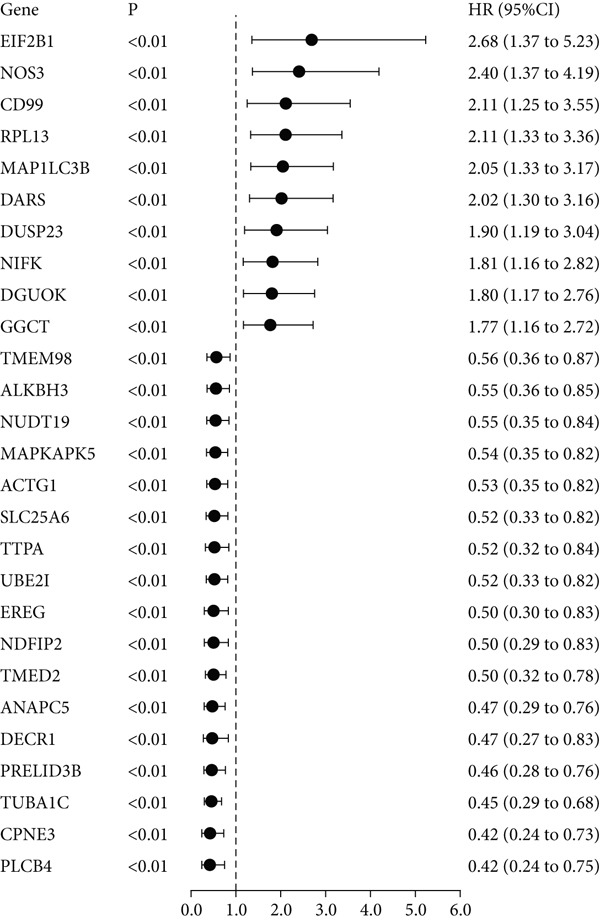
(a)

**Figure:**
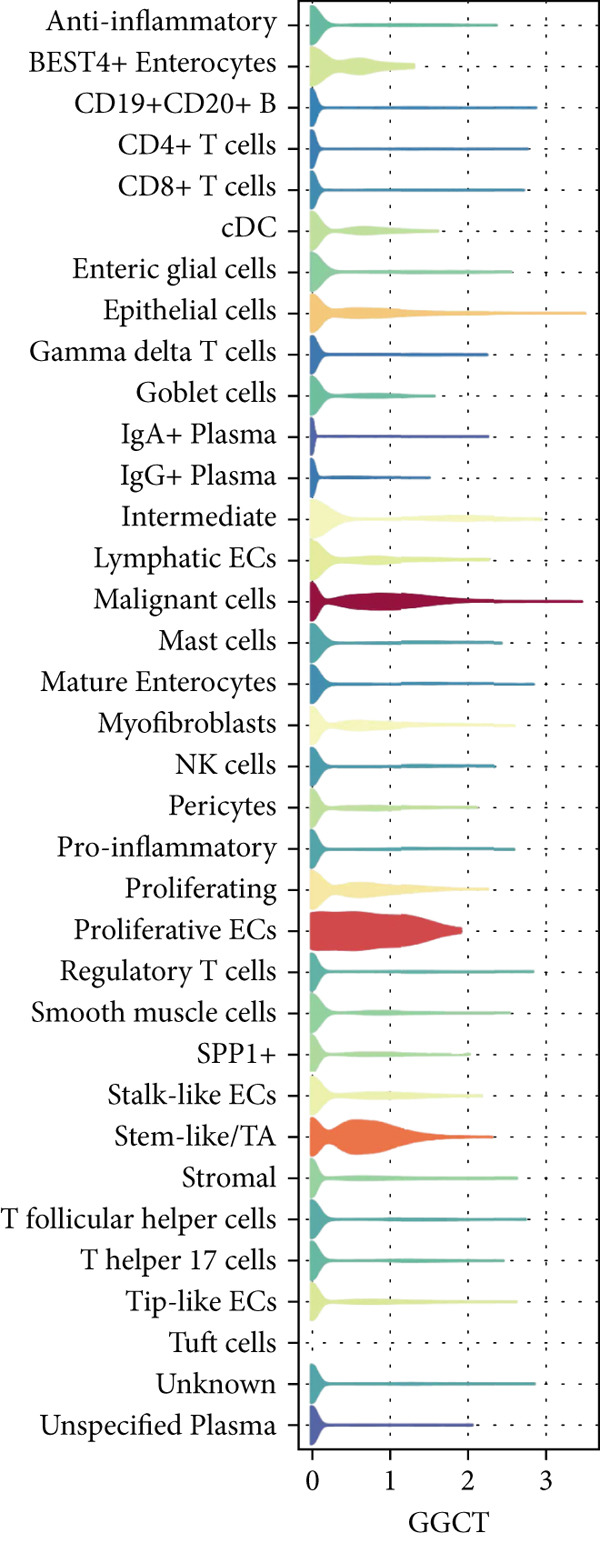
(b)

**Figure:**
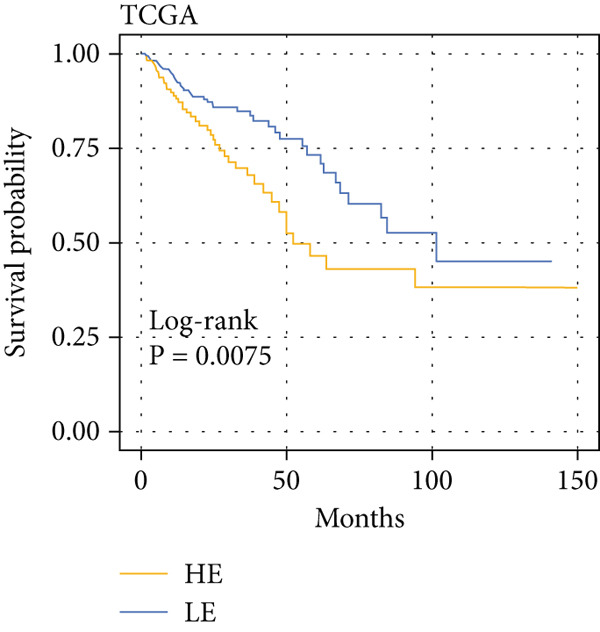
(c)

**Figure:**
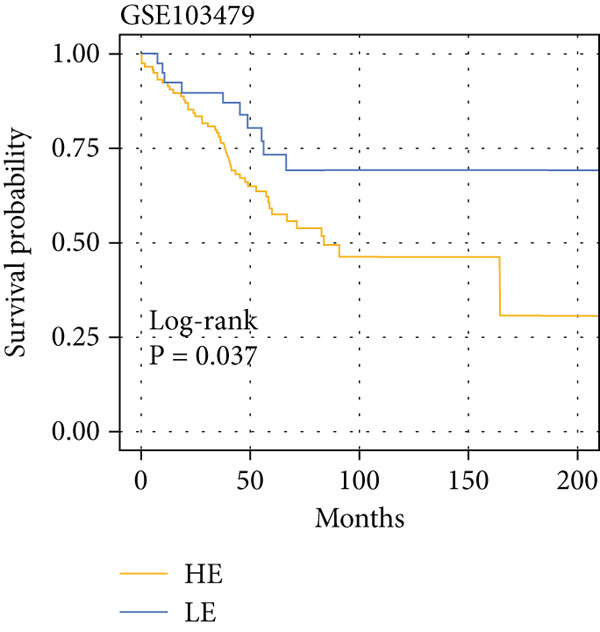
(d)

**Figure:**
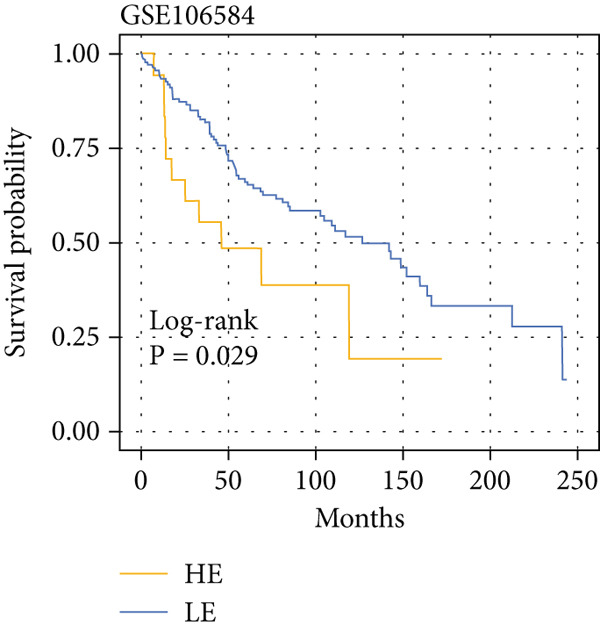
(e)

**Figure:**
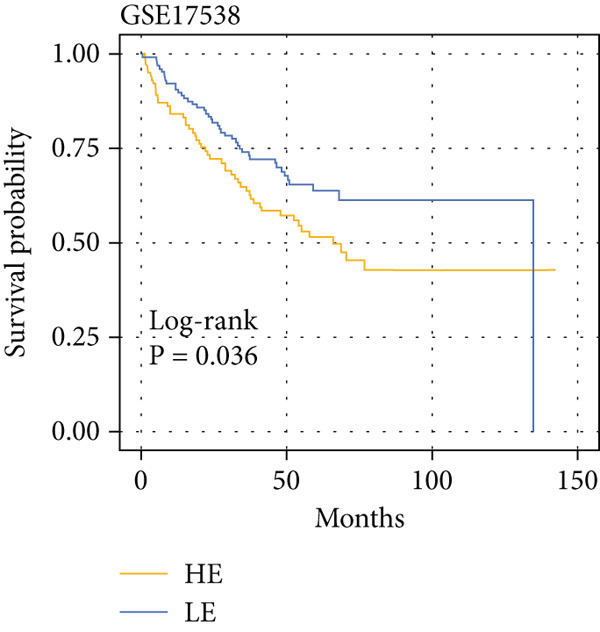
(f)

**Figure:**
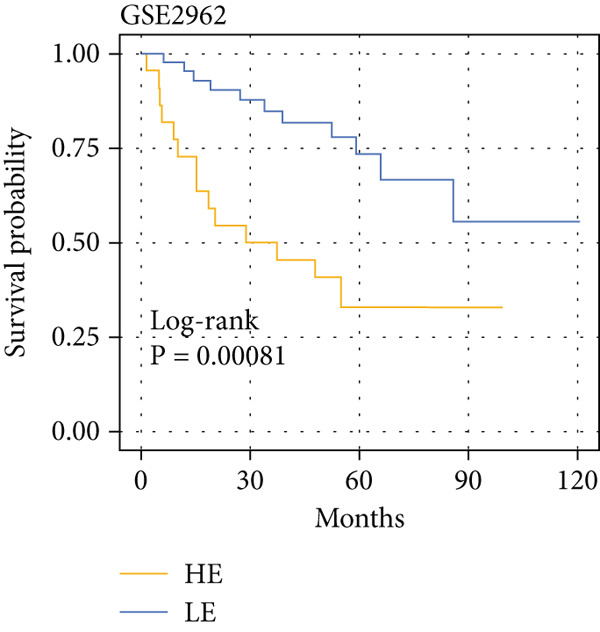
(g)

**Figure:**
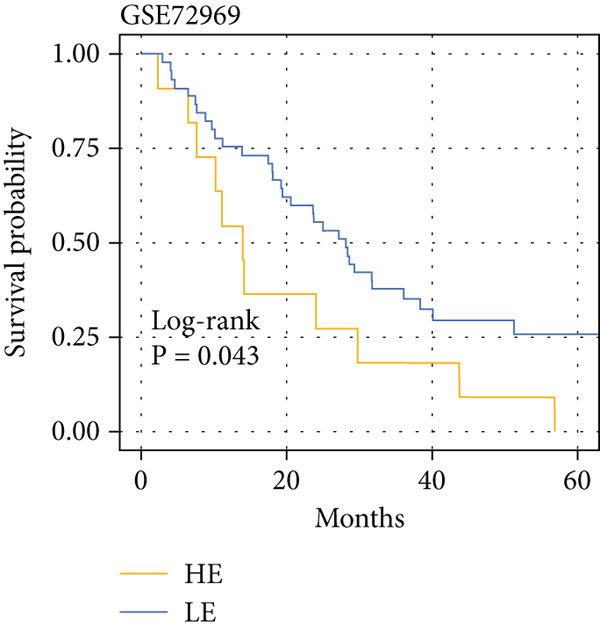
(h)

### 3.5. Functional Annotation of GGCT

**Figure 5:**
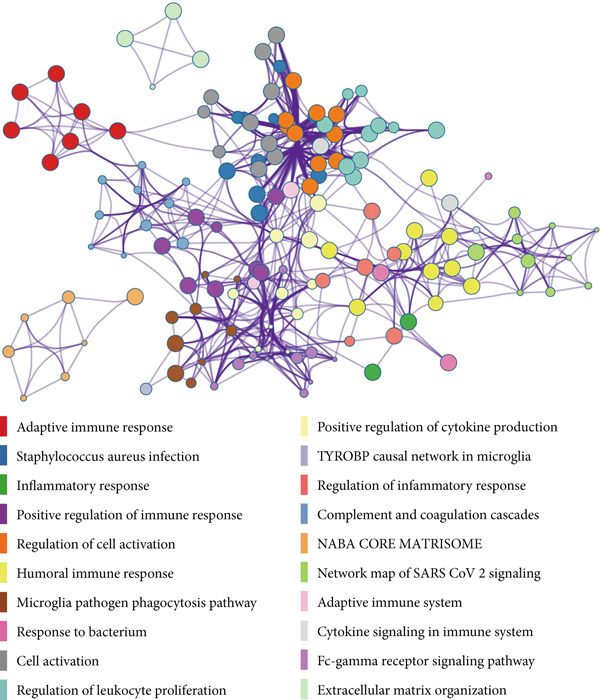
Functional annotation of GGCT. Bubble plot showing the Metascape‐based pathway enrichment analysis on DEGs between GGCT‐based groups.

To understand the biological pathways associated with GGCT, we performed differential gene expression analysis between high and low GGCT groups in the TCGA cohort and subjected the DEGs to pathway enrichment analysis using Metascape. The results, presented as a bubble plot in Figure 5, showed that GGCT‐high tumors were significantly enriched in pathways related to cell cycle regulation, DNA replication, immune regulation, and several oncogenic signaling pathways. This suggests that GGCT may promote CRC aggressiveness by driving proliferation and genomic instability.

### 3.6. GGCT Is Associated With an Immunosuppressive TME

Using the ESTIMATE algorithm, we found that tumors with high GGCT expression had significantly higher stromal, immune, and ESTIMATE scores, but lower tumor purity (Figure 6a), indicating a greater abundance of nontumor cellular components. Further analysis using TIMER revealed that GGCT‐high tumors had significantly altered immune infiltration, characterized by a decrease in CD8+ T cells and dendritic cells (DCs) and an increase in immunosuppressive macrophages (Figure 6b). This profile is consistent with an immune‐excluded or immunosuppressive TME, which may facilitate immune escape.

Figure 6Immune features of GGCT. (a) The levels of stromal score, immune score, and ESTIMATE score in GGCT‐based groups. (b) The abundance of B cells, CD4 T cells, CD8 T cells, neutrophils, macrophages, and DCs in GGCT‐based groups.

**Figure:**
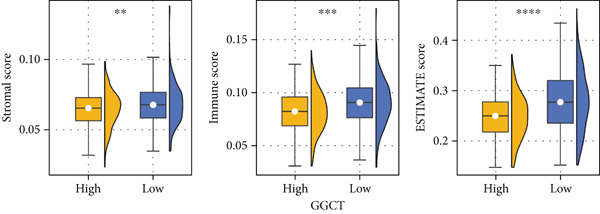
(a)

**Figure:**
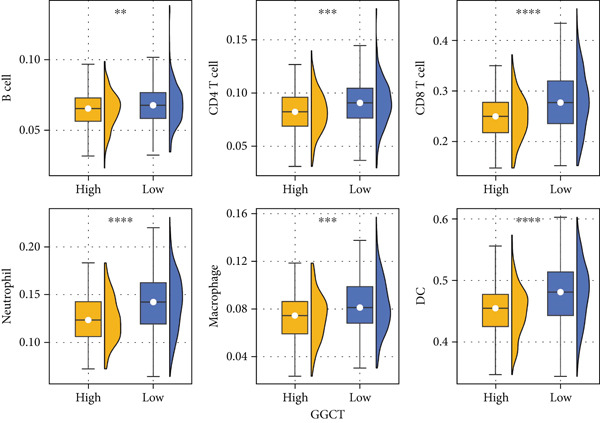
(b)

### 3.7. GGCT Predicts Immunotherapy Response

**Figure 7:**
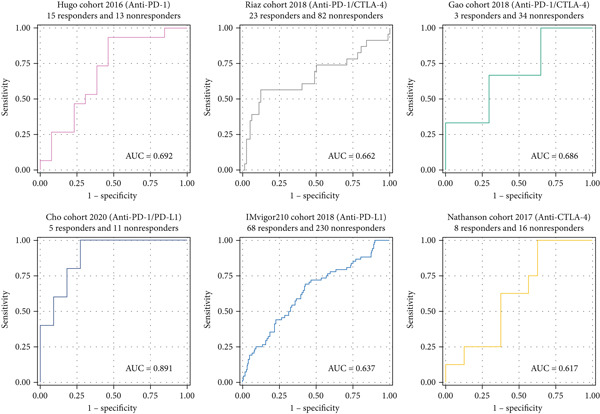
Immunotherapy predictive value of GGCT in six cohorts (Hugo cohort, Riaz cohort, Gao cohort, Cho cohort, IMvigor210 cohort, and Nathanson cohort).

Given its association with immune evasion, we evaluated the predictive value of GGCT for immunotherapy response in six publicly available cohorts of patients treated with immune checkpoint inhibitors (Hugo, Riaz, Gao, Cho, IMvigor210, and Nathanson). In most of these cohorts, patients with high GGCT expression had significantly lower rates of clinical response (e.g., complete or partial response) and poorer survival after immunotherapy (Figure 7), suggesting that GGCT could serve as a novel predictive biomarker for resistance to immunotherapy.

## 4. Discussion

CRC is the world′s fourth most deadly cancer, with almost 900,000 deaths annually [5]. The high mortality rate is predominantly attributable to metastasis [28, 29], which is often facilitated by IE. This study presents a comprehensive analysis of IE‐related genes in CRC, leveraging both single‐cell and bulk transcriptomic data. We constructed a detailed cellular map of the CRC TME, identified a key IE‐related gene module through hdWGCNA, and validated GGCT as a novel and robust prognostic biomarker across multiple independent cohorts. Furthermore, we elucidated its functional roles in promoting cell proliferation and shaping an immunosuppressive TME and demonstrated its potential value in predicting response to immunotherapy.

Our scRNA‐seq analysis confirmed the profound heterogeneity of the CRC TME, consistent with previous studies. The identification of GGCT as predominantly expressed in malignant cells aligns with its known role in other cancers; GGCT is involved in glutathione metabolism [30], which can protect cells from oxidative stress and confer chemoresistance. However, its specific role in immune modulation within CRC is novel. The consistent association between high GGCT expression and poor survival across all six bulk cohorts underscores its clinical relevance as a prognostic factor.

The functional enrichment analysis suggests that GGCT is not merely a passive marker but an active contributor to tumor progression. Its association with cell cycle and DNA replication pathways indicates a role in enhancing tumor cell proliferation. More importantly, the strong correlation between GGCT expression and an immunosuppressive TME landscape provides a mechanistic link to immune evasion. The observed reduction in cytotoxic CD8+ T cells and increase in protumor macrophages are hallmarks of a noninflamed TME, which is typically resistant to immunotherapy [31].

The predictive value of GGCT for immunotherapy resistance is a significant finding. Although biomarkers like PD‐L1 expression and tumor mutational burden are currently used [32, 33], they have limitations. GGCT offers a new, complementary perspective rooted in cellular metabolism and immune contexture. Its ability to predict worse outcomes after immunotherapy in multiple cohorts highlights its potential utility in clinical decision‐making, perhaps for patient stratification or combination therapy design.

Several limitations should be considered. This is a retrospective bioinformatics study based on public data. Experimental validation using in vitro and in vivo models is necessary to confirm the functional role of GGCT in immune evasion. The precise molecular mechanisms by which GGCT influences immune cell infiltration and function require further investigation, potentially involving interactions with specific cytokines or metabolic pathways.

In conclusion, our integrated multiomics approach identifies GGCT as a critical player in CRC immune evasion and prognosis. It impacts the TME by fostering an immunosuppressive milieu and is associated with resistance to immunotherapy. These findings nominate GGCT as a promising therapeutic target. Future work should focus on developing small‐molecule inhibitors against GGCT and testing their efficacy, alone or in combination with immunotherapy, in preclinical models of CRC.

## Ethics Statement

Ethics approval was not required for this study, as it analyzed existing genomic datasets with no direct involvement of humans or animals.

## Disclosure

S.Z. was responsible for the in vitro experiments.

## Conflicts of Interest

The authors declare no conflicts of interest.

## Author Contributions

Q.N., S.Y., and C.S. designed the specific details of this study and drafted the manuscript. Y.L. participated in data analysis, whereas K.H. and L.N. contributed to manuscript revisions.

## Funding

This study was supported by the Key Research and Development Program Project of Heilongjiang Province (2022ZX06C22), Heart to Heart Foundation (HXXT2021ktyj002), Wu Jieping Medical Foundation (10.13039/100007452; 320.6750.2023‐07‐6, and Beijing Medical Award Foundation (YXJL‐2021‐0763‐0482).

## Supporting information


**Supporting Information** Additional supporting information can be found online in the Supporting Information section.. Figure S1: Functional annotation of GGCT.

## Data Availability

The CRC single‐cell transcriptomic data were downloaded from the Gene Expression Omnibus (GEO), including GSE132465 and GSE14473514. The CRC bulk transcriptome data were downloaded from The Cancer Genome Atlas (TCGA) and the Gene Expression Omnibus (GEO), including COADREAD_TCGA, GSE106584, GSE29621, GSE103479, GSE17538, and GSE72969. All data from this study are available from the corresponding author upon reasonable request.

## References

[1] Ahn H. M. , Lee T. G. , Shin H. R. , Lee J. , Yang I. J. , Suh J. W. , Oh H. K. , Kim D. W. , and Kang S. B. , Oncologic Impact of Technical Difficulties During the Early Experience With Laparoscopic Surgery for Colorectal Cancer: Long-Term Follow-Up Results of a Prospective Cohort Study, Current Problems in Surgery. (2025) 63, 101694, 10.1016/j.cpsurg.2024.101694. 39922625

[2] Biller L. H. and Schrag D. , Diagnosis and Treatment of Metastatic Colorectal Cancer, Jama. (2021) 325, no. 7, 669–685, 10.1001/jama.2021.0106. 33591350

[3] Li Q. , Lu Y. , and Wang D. , Differential Survival Outcomes and Prognostic Factors Across Surgical Approaches for Colorectal Cancer: Analysis of 5000 Patients from the Seer Database, Current Problems in Surgery. (2025) 70, 101839, 10.1016/j.cpsurg.2025.101839. 40812984

[4] Shen C. , Li W. , Shi J. , Chen L. , Ning N. , and Yan Y. , Comparative Prognostic Value of Ultrasound and CT Scan in Postoperative Follow-Up Of Colorectal Cancer, Current Problems in Surgery. (2025) 69, 101786, 10.1016/j.cpsurg.2025.101786. 40716888

[5] Dekker E. , Tanis P. J. , Vleugels J. L. A. , Kasi P. M. , and Wallace M. B. , Colorectal Cancer, Lancet. (2019) 394, no. 10207, 1467–1480, 10.1016/S0140-6736(19)32319-0, 2-s2.0-85073506559. 31631858

[6] Gu S. , Chen S. , Chai Y. , Qu C. , Sun X. , and Yu J. , Predictive Value of Preoperative Serum Cytokeratin 19 Fragment Antigen 21-1(CYFRA 21-1) in Surgical Resection for Colorectal Cancer: A Retrospective Study, Current Problems in Surgery. (2025) 69, 101791, 10.1016/j.cpsurg.2025.101791. 40716848

[7] Belisa T. K. , Zewde E. B. , Deress A. S. , Belay H. A. , Abrar H. T. , and Belay I. M. , Young-Onset Colorectal Cancer Complicated by Acute Ischemic Stroke: A Comprehensive Case Report And Literature Review, Current Problems in Surgery. (2025) 68, 101768, 10.1016/j.cpsurg.2025.101768. 40500028

[8] Huang M. , Qin B. , Xie Z. , Zhou R. , Lin Z. , Zhou L. , Han Z. , Liu S. , and Zhuang K. , RCN3 Functions as a Tumor Promoter in Colorectal Cancer by Modulating the GRP78-PI3K-AKT Signaling Pathway, Oncogene. (2025) 44, no. 40, 3850–3863, 10.1038/s41388-025-03535-5. 40849588

[9] Chevrier S. , Levine J. H. , Zanotelli V. R. T. , Silina K. , Schulz D. , Bacac M. , Ries C. H. , Ailles L. , Jewett M. A. S. , Moch H. , van den Broek M. , Beisel C. , Stadler M. B. , Gedye C. , Reis B. , Pe′er D. , and Bodenmiller B. , An Immune Atlas of Clear Cell Renal Cell Carcinoma, Cell. (2017) 169, no. 4, 736–749.e18, 10.1016/j.cell.2017.04.016, 2-s2.0-85018787063, 28475899. 28475899 PMC5422211

[10] Wang X. , Lopez R. , Luchtel R. A. , Hafizi S. , Gartrell B. , and Shenoy N. , Immune Evasion in Renal Cell Carcinoma: Biology, Clinical Translation, Future Directions, Kidney International. (2021) 99, no. 1, 75–85, 10.1016/j.kint.2020.08.028. 32949550

[11] Zhang N. , Yang M. , Yang J. M. , Zhang C. Y. , and Guo A. Y. , A Predictive Network‐Based Immune Checkpoint Blockade Immunotherapeutic Signature Optimizing Patient Selection and Treatment Strategies, Small Methods. (2024) 8, no. 10, e2301685, 10.1002/smtd.202301685. 38546036

[12] Li S. , Zhang N. , Zhang H. , Yang Z. , Cheng Q. , Wei K. , Zhou M. , and Huang C. , Deciphering the Role of LGALS2: Insights Into Tertiary Lymphoid Structure-Associated Dendritic Cell Activation and Immunotherapeutic Potential in Breast Cancer Patients, Molecular Cancer. (2024) 23, no. 1, 10.1186/s12943-024-02126-4, 39350165. PMC11441145 39350165

[13] Tang F. , Li J. , Qi L. , Liu D. , Bo Y. , Qin S. , Miao Y. , Yu K. , Hou W. , Li J. , Peng J. , Tian Z. , Zhu L. , Peng H. , Wang D. , and Zhang Z. , A Pan-Cancer Single-Cell Panorama of Human Natural Killer Cells, Cell. (2023) 186, no. 19, 4235–4251.e20, 10.1016/j.cell.2023.07.034, 37607536. 37607536

[14] Lee H. O. , Hong Y. , Etlioglu H. E. , Cho Y. B. , Pomella V. , Van den Bosch B. , Vanhecke J. , Verbandt S. , Hong H. , Min J. W. , Kim N. , Eum H. H. , Qian J. , Boeckx B. , Lambrechts D. , Tsantoulis P. , De Hertogh G. , Chung W. , Lee T. , An M. , Shin H. T. , Joung J. G. , Jung M. H. , Ko G. , Wirapati P. , Kim S. H. , Kim H. C. , Yun S. H. , Tan I. B. H. , Ranjan B. , Lee W. Y. , Kim T. Y. , Choi J. K. , Kim Y. J. , Prabhakar S. , Tejpar S. , and Park W. Y. , Lineage-Dependent Gene Expression Programs Influence the Immune Landscape of Colorectal Cancer, Nature Genetics. (2020) 52, no. 6, 594–603, 10.1038/s41588-020-0636-z. 32451460

[15] Satija R. , Farrell J. A. , Gennert D. , Schier A. F. , and Regev A. , Spatial Reconstruction of Single-Cell Gene Expression Data, Nature Biotechnology. (2015) 33, no. 5, 495–502, 10.1038/nbt.3192, 2-s2.0-84929151009, 25867923. PMC4430369 25867923

[16] Barbie D. A. , Tamayo P. , Boehm J. S. , Kim S. Y. , Moody S. E. , Dunn I. F. , Schinzel A. C. , Sandy P. , Meylan E. , Scholl C. , Frohling S. , Chan E. M. , Sos M. L. , Michel K. , Mermel C. , Silver S. J. , Weir B. A. , Reiling J. H. , Sheng Q. , Gupta P. B. , Wadlow R. C. , Le H. , Hoersch S. , Wittner B. S. , Ramaswamy S. , Livingston D. M. , Sabatini D. M. , Meyerson M. , Thomas R. K. , Lander E. S. , Mesirov J. P. , Root D. E. , Gilliland D. G. , Jacks T. , and Hahn W. C. , Systematic RNA Interference Reveals that Oncogenic KRAS-Driven Cancers Require TBK1, Nature. (2009) 462, no. 7269, 108–112, 10.1038/nature08460, 2-s2.0-70449091786, 19847166. 19847166 PMC2783335

[17] Zhu J. , Deane N. G. , Lewis K. B. , Padmanabhan C. , Washington M. K. , Ciombor K. K. , Timmers C. , Goldberg R. M. , Beauchamp R. D. , and Chen X. , Evaluation of Frozen Tissue-Derived Prognostic Gene Expression Signatures in FFPE Colorectal Cancer Samples, Scientific Reports. (2016) 6, 33273, 10.1038/srep33273, 2-s2.0-84987849194, 27623752. 27623752 PMC5021945

[18] Chen D. T. , Hernandez J. M. , Shibata D. , McCarthy S. M. , Humphries L. A. , Clark W. , Elahi A. , Gruidl M. , Coppola D. , and Yeatman T. , Complementary Strand Micrornas Mediate Acquisition of Metastatic Potential in Colonic Adenocarcinoma, Journal of Gastrointestinal Surgery. (2012) 16, no. 5, 905–912, 10.1007/s11605-011-1815-0, 2-s2.0-84865324333, 22362069. 22362069 PMC6753785

[19] Allen W. L. , Dunne P. D. , McDade S. , Scanlon E. , Loughrey M. , Coleman H. , McCann C. , McLaughlin K. , Nemeth Z. , Syed N. , Jithesh P. , Arthur K. , Wilson R. , Coyle V. , McArt D. , Murray G. I. , Samuel L. , Nuciforo P. , Jimenez J. , Argiles G. , Dienstmann R. , Tabernero J. , Messerini L. , Nobili S. , Mini E. , Sheahan K. , Ryan E. , Johnston P. G. , Van Schaeybroeck S. , Lawler M. , and Longley D. B. , Transcriptional Subtyping and CD8 Immunohistochemistry Identifies Poor Prognosis Stage II/III Colorectal Cancer Patients Who Benefit From Adjuvant Chemotherapy, JCO Precision Oncology. (2018) 2, 1–15, 10.1200/PO.17.00241, 30088816. PMC6040635 30088816

[20] Smith J. J. , Deane N. G. , Wu F. , Merchant N. B. , Zhang B. , Jiang A. , Lu P. , Johnson J. C. , Schmidt C. , Bailey C. E. , Eschrich S. , Kis C. , Levy S. , Washington M. K. , Heslin M. J. , Coffey R. J. , Yeatman T. J. , Shyr Y. , and Beauchamp R. D. , Experimentally Derived Metastasis Gene Expression Profile Predicts Recurrence and Death in Patients With Colon Cancer, Gastroenterology. (2010) 138, no. 3, 958–968, 10.1053/j.gastro.2009.11.005, 2-s2.0-77249143960, 19914252. 19914252 PMC3388775

[21] Del Rio M. , Mollevi C. , Bibeau F. , Vie N. , Selves J. , Emile J. F. , Roger P. , Gongora C. , Robert J. , Tubiana-Mathieu N. , Ychou M. , and Martineau P. , Molecular Subtypes of Metastatic Colorectal Cancer are Associated with Patient Response to Irinotecan-Based Therapies, European Journal of Cancer. (2017) 76, 68–75, 10.1016/j.ejca.2017.02.003, 2-s2.0-85014633340. 28284171

[22] Jia H. R. , Li W. C. , and Wu L. , The Prognostic Value of Immune Escape-Related Genes in Lung Adenocarcinoma, Translational Cancer Research. (2024) 13, no. 6, 2647–2661, 10.21037/tcr-23-2295, 38988926. 38988926 PMC11231773

[23] Morabito S. , Reese F. , Rahimzadeh N. , Miyoshi E. , and Swarup V. , hdWGCNA Identifies Co-Expression Networks in High-Dimensional Transcriptomics Data, Cell Reports Methods. (2023) 3, no. 6, 100498, 10.1016/j.crmeth.2023.100498, 37426759. 37426759 PMC10326379

[24] Maeser D. , Gruener R. F. , and Huang R. S. , oncoPredict: An R Package for Predicting *in Vivo* or Cancer Patient Drug Response and Biomarkers From Cell Line Screening Data, Briefings in Bioinformatics. (2021) 22, no. 6, 10.1093/bib/bbab260, bbab260, 34260682. 34260682 PMC8574972

[25] Davoli T. , Uno H. , Wooten E. C. , and Elledge S. J. , Tumor Aneuploidy Correlates With Markers of Immune Evasion and With Reduced Response to Immunotherapy, Science. (2017) 355, no. 6322, 10.1126/science.aaf8399, 2-s2.0-85010652953, eaaf8399, 28104840. 28104840 PMC5592794

[26] Yoshihara K. , Shahmoradgoli M. , Martínez E. , Vegesna R. , Kim H. , Torres-Garcia W. , Treviño V. , Shen H. , Laird P. W. , Levine D. A. , Carter S. L. , Getz G. , Stemke-Hale K. , Mills G. B. , and Verhaak R. G. W. , Inferring Tumour Purity and Stromal and Immune Cell Admixture From Expression Data, Nature Communications. (2013) 4, no. 1, 10.1038/ncomms3612, 2-s2.0-84885673911, 24113773. PMC3826632 24113773

[27] Zhou Y. , Zhou B. , Pache L. , Chang M. , Khodabakhshi A. H. , Tanaseichuk O. , Benner C. , and Chanda S. K. , Metascape Provides A Biologist-Oriented Resource for the Analysis of Systems-Level Datasets, Nature Communications. (2019) 10, no. 1, 10.1038/s41467-019-09234-6, 2-s2.0-85063948265, 30944313. PMC6447622 30944313

[28] Yu W. , Lin Y. , Huang M. , Du Y. , and Ye Z. , The Connection Between Surgical Margins in Liver Metastases From Colorectal Cancer and Patient Prognosis: A Mini Review, Current Problems in Surgery. (2025) 63, 101700, 10.1016/j.cpsurg.2024.101700. 39922639

[29] Liu J. , Jiang P. , Zhang Z. , Yang H. , Zhou Y. , Li P. , Zeng Q. , Zhang X. , and Sun Y. , Survival Analysis in Rectal Cancer Patients After Lateral Lymph Node Dissection: Exploring the Necessity of nCRT for Suspected Lateral Lymph Node Metastasis, Current Problems in Surgery. (2024) 61, no. 8, 101525, 10.1016/j.cpsurg.2024.101525. 39098341

[30] Kageyama S. , Ii H. , Taniguchi K. , Kubota S. , Yoshida T. , Isono T. , Chano T. , Yoshiya T. , Ito K. , Yoshiki T. , Kawauchi A. , and Nakata S. , Mechanisms of Tumor Growth Inhibition by Depletion of *γ*-Glutamylcyclotransferase (GGCT): A Novel Molecular Target for Anticancer Therapy, International Journal of Molecular Sciences. (2018) 19, no. 7, 10.3390/ijms19072054, 2-s2.0-85050244980, 30011933. PMC6073726 30011933

[31] Bejarano L. , Jordao M. J. C. , and Joyce J. A. , Therapeutic Targeting of the Tumor Microenvironment, Cancer Discovery. (2021) 11, no. 4, 933–959, 10.1158/2159-8290.CD-20-1808. 33811125

[32] Lee D. , Cho M. , Kim E. , Seo Y. , and Cha J. H. , PD-L1: From Cancer Immunotherapy to Therapeutic Implications in Multiple Disorders, Molecular Therapy. (2024) 32, no. 12, 4235–4255, 10.1016/j.ymthe.2024.09.026, 39342430. 39342430 PMC11638837

[33] Zhang N. , Zhang H. , Li S. , Wu W. , Luo P. , Liu Z. , Chen Y. , Xia Z. , Huang C. , and Cheng Q. , Uncovering the Predictive and Immunomodulatory Potential of Transient Receptor Potential Melastatin Family-Related CCNE1 in Pan-Cancer, Molecular Cancer. (2024) 23, no. 1, 10.1186/s12943-024-02169-7, 39551726. PMC11572178 39551726

