# Supplementary figures and images for: Integrative Multiomics Nominate GGCT as a Crucial Regulator of Immunosuppression in Colorectal Cancer
Source: Int J Genomics. 2026 Jan 21;2026:7013449. doi: 10.1155/ijog/7013449 (PMC12820795; doi:10.1155/ijog/7013449)

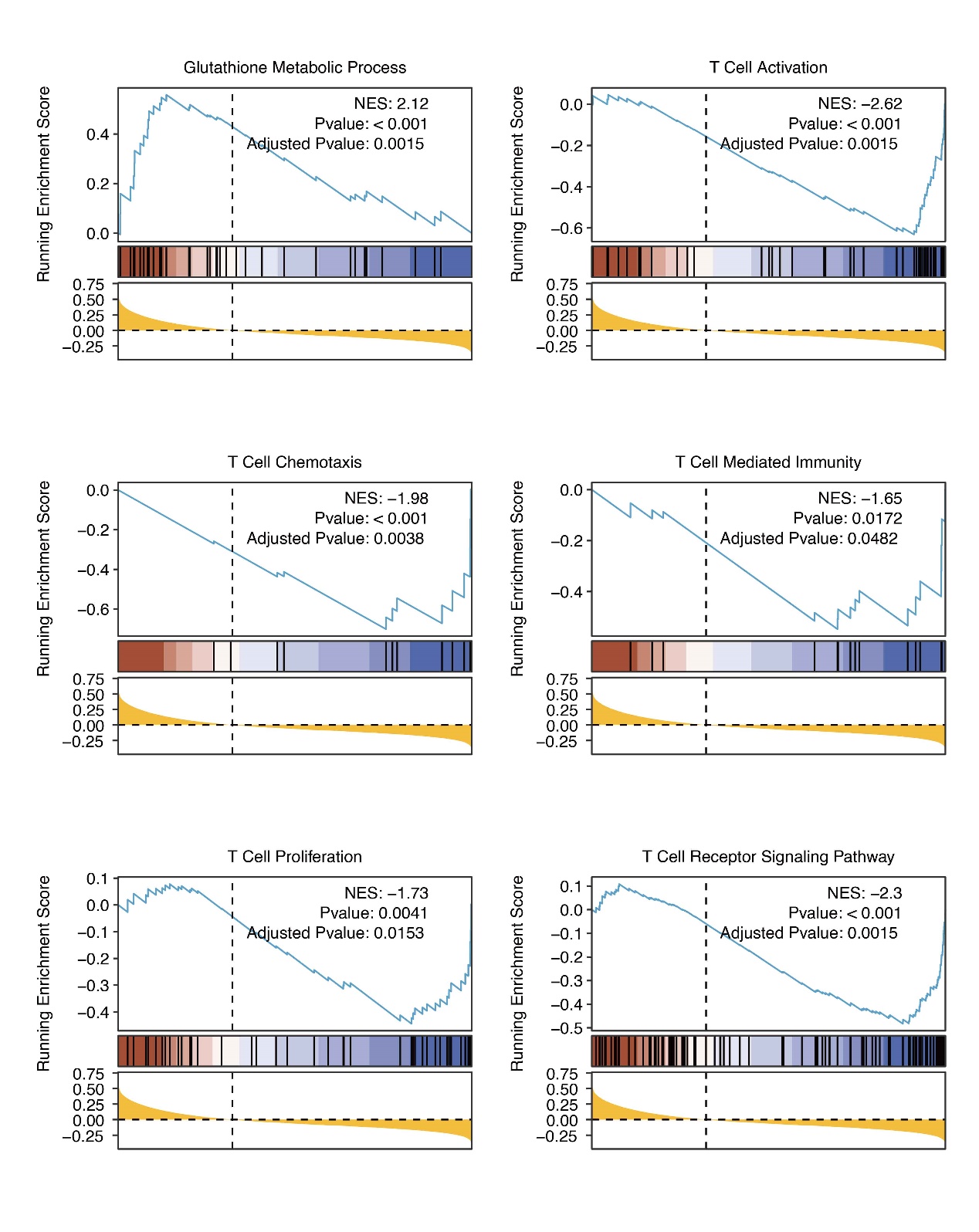


Figure S1. Functional annotation of GGCT.


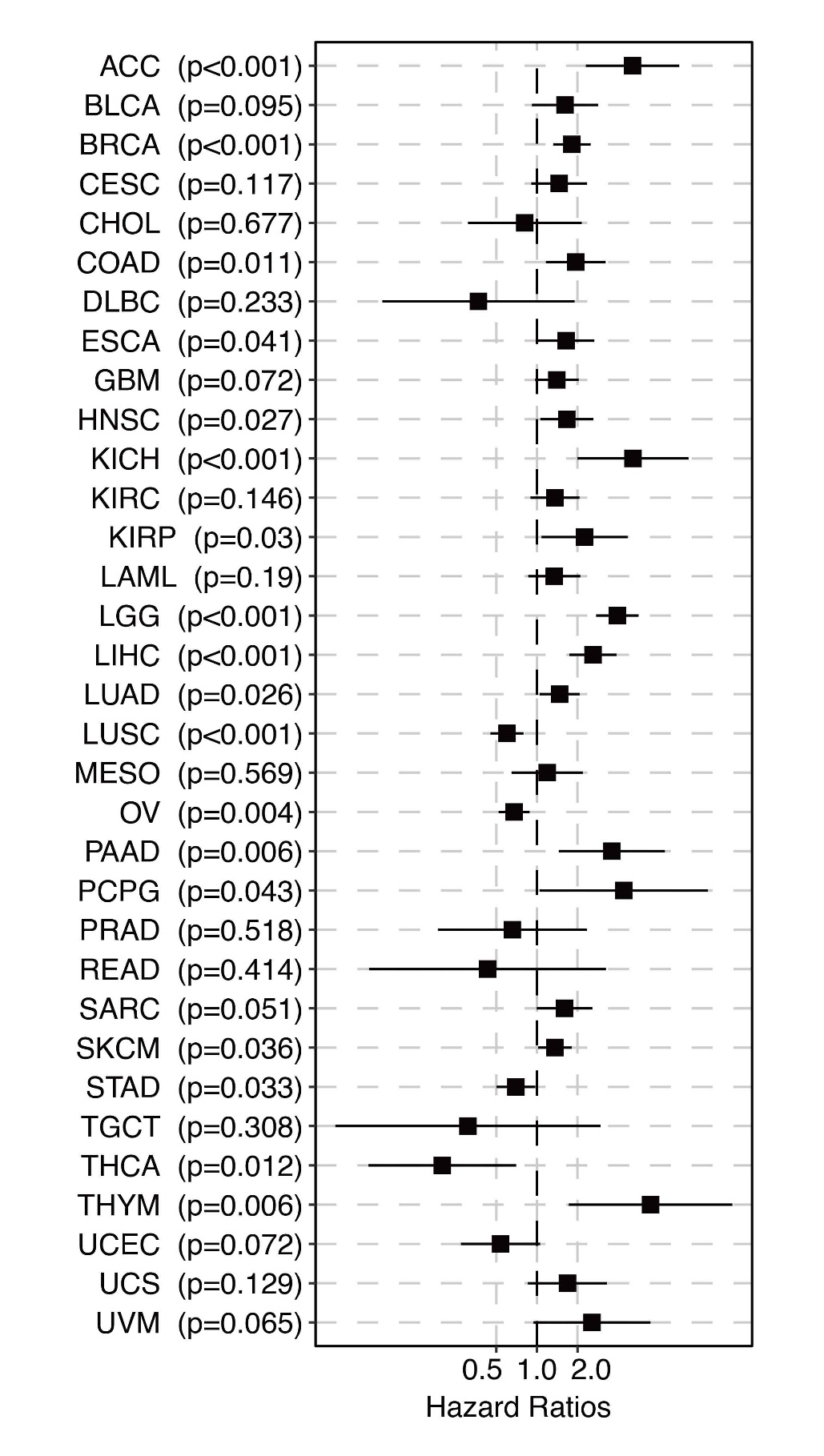


Figure S2. Pan-cancer role of GGCT.

Supplement: Supplementary file 1 — Supporting Information Additional supporting information can be found online in the Supporting Information section.. Figure S1: Functional annotation of GGCT. [file IJOG-2026-7013449-s001.docx]
